# Relative contributions of oculomotor capture and disengagement to distractor-related dwell times in visual search

**DOI:** 10.1038/s41598-023-43604-x

**Published:** 2023-10-04

**Authors:** Maximilian Stefani, Marian Sauter

**Affiliations:** 1 Institute of Psychology, General Psychology, Bundeswehr University Munich, Werner-Heisenberg-Weg, 39, 85577 Neubiberg, Germany; 2 Institute of Psychology, General Psychology, Ulm University, 89069 Ulm, Germany

**Keywords:** Psychology, Human behaviour

## Abstract

In visual search, attention is reliably captured by salient distractors and must be actively disengaged from them to reach the target. In such attentional capture paradigms, dwell time is measured on distractors that appear in the periphery (e.g., on a random location on a circle). Distractor-related dwell time is typically thought to be largely due to stimulus-driven processes related to oculomotor capture dynamics. However, the extent to which oculomotor capture and oculomotor disengagement contribute to distractor dwell time has not been known because standard attentional capture paradigms cannot decouple these processes. In the present study, we used a novel paradigm combining classical attentional capture trials and delayed disengagement trials. We measured eye movements to dissociate the capture and disengagement mechanisms underlying distractor dwell time. We found that only two-thirds of distractor dwell time (~ 52 ms) can be explained by oculomotor capture, while one-third is explained by oculomotor disengagement (~ 18 ms), which has been neglected or underestimated in previous studies. Thus, oculomotor disengagement (goal-directed) processes play a more significant role in distractor dwell times than previously thought.

## Introduction

When looking for something in a busy environment, other important things (distractors) can get in the way of finding the target. Some research shows that a cognitive suppression mechanism can, in many circumstances, prevent these distractors from getting or 'capturing' attention^1–5^, but other times it fails, the distractors are noticed^1,6–9^ and maybe reactively suppressed (in order to disengage from the distractor item, the attentional priority is downmodulated reactively, i.e., after attention has already shifted to the distractor)^7,10,11^. Whenever our attention is (involuntarily) allocated to a salient distractor before it is re-allocated to the target, we might either move on to the next important item without additional cognitive effort (this means that there are now time costs attributed to these processes)^12,13^ or the distractor has to be actively suppressed before we can move on. The average difference in response time for stimulus displays that feature such a salient distractor (attention often gets allocated to the distractor before the target) versus stimulus displays that do not feature such a distractor (attention is often directly allocated to the target) is referred to as *distractor cost* or *distractor interference*.

The way we decide which object to pay attention to next is hypothesized based on a cognitive *priority map*^14–18^. This map is determined by weighting and combining what we see (stimulus features) and what we want to find (task goals). Distractors can capture our attention (and are thereby attended involuntarily) if valued higher on this map than the target object^1,6,8,9,19–21^. Even if the target is less important at first, it will (usually) eventually be found, and the correct action can be initiated. Such a basic priority map concept leaves room for two ways of how attentional capture can occur that led to a 30-year-long debate: First, stimuli that are perceived as sudden or stand out from their surroundings often attract attention, referred to as *salient*^22^. Attention is automatically drawn to these unique features regardless of task relevance^23–26^. Such accounts are described as *stimulus-driven* or *bottom-up* because the interference is generated at the bottom (stimulus level) in the cognitive hierarchy and affects levels in an upwards fashion^27^. Second, these concepts of goal-independent attentional capture by salient stimuli were challenged by the contingent involuntary orienting hypothesis^28^, which posits that attention will be drawn to a stimulus only if it aligns with an attentional set. These attentional sets are task-dependent, such as a search for a particular shape singleton, which may prime attention towards all singletons, including irrelevant color singletons^21,29^. Such hypotheses are referred to as *goal-driven* or *top-down* because the interference is generated at the top of the cognitive hierarchy and affects levels downwards^18,30^. The disagreement between the two accounts has resulted in a long-standing debate, with the contingent involuntary orienting hypothesis^31,32^ and stimulus-driven attention^33–35^ garnering support from various studies.

More recently, the dialogue has shifted due to increasing evidence of an inhibitory process that can counteract attentional capture, even when a stimulus emits a priority signal^36–40^. The current understanding of attentional capture by physical salience highlights that salient stimuli can trigger a priority signal that will capture attention in the absence of inhibitory mechanisms. Nevertheless, there exist conditions under which the actual capturing of attention can be prevented. So, both goal-driven and stimulus-driven factors may contribute to attentional capture effects at the same time. In eye-tracking studies, two measurements are typically used to evaluate attentional capture. The first, and perhaps most prevalent, is the landing position of the initial saccades, which reflects the prevalence of capture—that is, the proportion of first saccades after display onset that land on the distractor item. The second measure, which is the focus of our study, is the distractor dwell time, which is the time the eyes are fixated on the distractor item. This measure gives us an estimate of the size of attentional capture effects in terms of the additional time cost (capture costs). This was found to be around ~ 100 ms^41–46^. From the ongoing debate, *selection history*, which refers to the influence of past experiences and learned associations on the allocation of attention, emerged as the third dominant factor influencing attentional capture^47–50^ and has since extended stimulus-driven and goal-driven models while also providing a joint basis for progress towards resolving the debate^51^.

Despite extensive study on the sources of attentional capture, the distinction between the interference associated with a distractor's ability to direct our gaze to a location (oculomotor capture) and the interference associated with its ability to keep attention at a location once it has been allocated (oculomotor disengagement) was rarely made. Both calculations rely on the distractor dwell time in classical attentional capture paradigms. Therefore, it is difficult to separate how much distractor dwell time pertains to the capture and how much to the disengagement processes. This distinction is crucial because memory and other human experiences are influenced by the locations to which attention is directed and the duration of attention at each location^52,53^. For example, research indicates that attentional disengagement is delayed as the object in focus becomes increasingly similar to the target^27,54^. Thus, understanding the factors that impact attentional disengagement is crucial for comprehending capture and developing comprehensive models of visual search. Several studies^55–57^ have explored this issue directly by examining the ability of task-irrelevant objects and features to hold overt attention. These studies employed the *disengagement paradigm*, which involved a search task in which participants began by fixating on a central item that could never be the target. A perceptual change to this item accompanied the target's appearance and distractors in the periphery. To complete the search, participants had to disengage from the central item and shift attention to the target in the periphery. The disengagement time was calculated as the interval between the search display's presentation and the gaze's departure from the central item (oculomotor disengagement time). With eye tracking, it was shown that oculomotor disengagement from central fixation items could be delayed under the circumstances^56,57^, which may result in (involuntarily) deeper processing of the fixation item^58^.

However, it remains unclear to which extent this distractor dwell time can be attributed to attentional capture or disengagement (extreme case: without any dwell time, we can neither compute the size of capture nor disengagement costs). Generally, it seems plausible that distractors interfere with the search for the target within two phases: (1) capture phase: the initial capture of attention, including allocation of attention to the distractor item and moving the eyes to the distractor item, and (2) disengagement phase: investigating the item, rejecting the item as a search target and moving on to the next item. Importantly, this does not imply that all capture-related processes happen only during the capture phase. In an attempt to relate capture and disengagement processes to the debate on goal-driven and stimulus-driven factors in attentional capture, Born and colleagues^59^ demonstrated that the mechanisms of oculomotor capture and disengagement can be distinct in the presence of salient onset distractors that trigger rapid capture saccades. Oculomotor capture seems primarily stimulus-driven and relies heavily on the earliest signals the oculomotor system receives^59^. Oculomotor disengagement is primarily controlled by top-down processes and functionally dissociable from capture. Additionally, in trials where the distractor shares features with the target, distractor dwell times increased from 80–90 ms to 120 ms. Nevertheless, changes in the distractor features that happened beyond 60–80 ms after the target onset did not alter oculomotor disengagement times, which was hypothesized to be due to the parallel programming of saccades. This implies that it is not the feedback signal from the currently fixated distractor that determines the distractor dwell time, but instead, the dwell time is crucially dependent on the initial distractor information. However, while it became apparent that the distractor dwell time is an essential factor in the overall attentional capture effect, it is still unclear to which extent this dwell time is due to oculomotor capture versus disengagement. These findings provide the crucial groundwork for our study. We aim to expand upon these insights by delving deeper into the attentional capture and disengagement processes, with a particular focus on the allocation of dwell time to each of these phases. The aim is to uncover the extent to which the dwell time can be attributed to either of these processes. In the study by Born and colleagues^59^, this could not be differentiated because the distractor dwell time is never investigated without the need for distractor-targeted saccades, thereby confounded by distractor-related saccade planning processes. This means that in all trials where we successfully capture a distractor item, the identification of the distractor as a target candidate and the saccade to this item needs to be planned.

**Figure 1:**
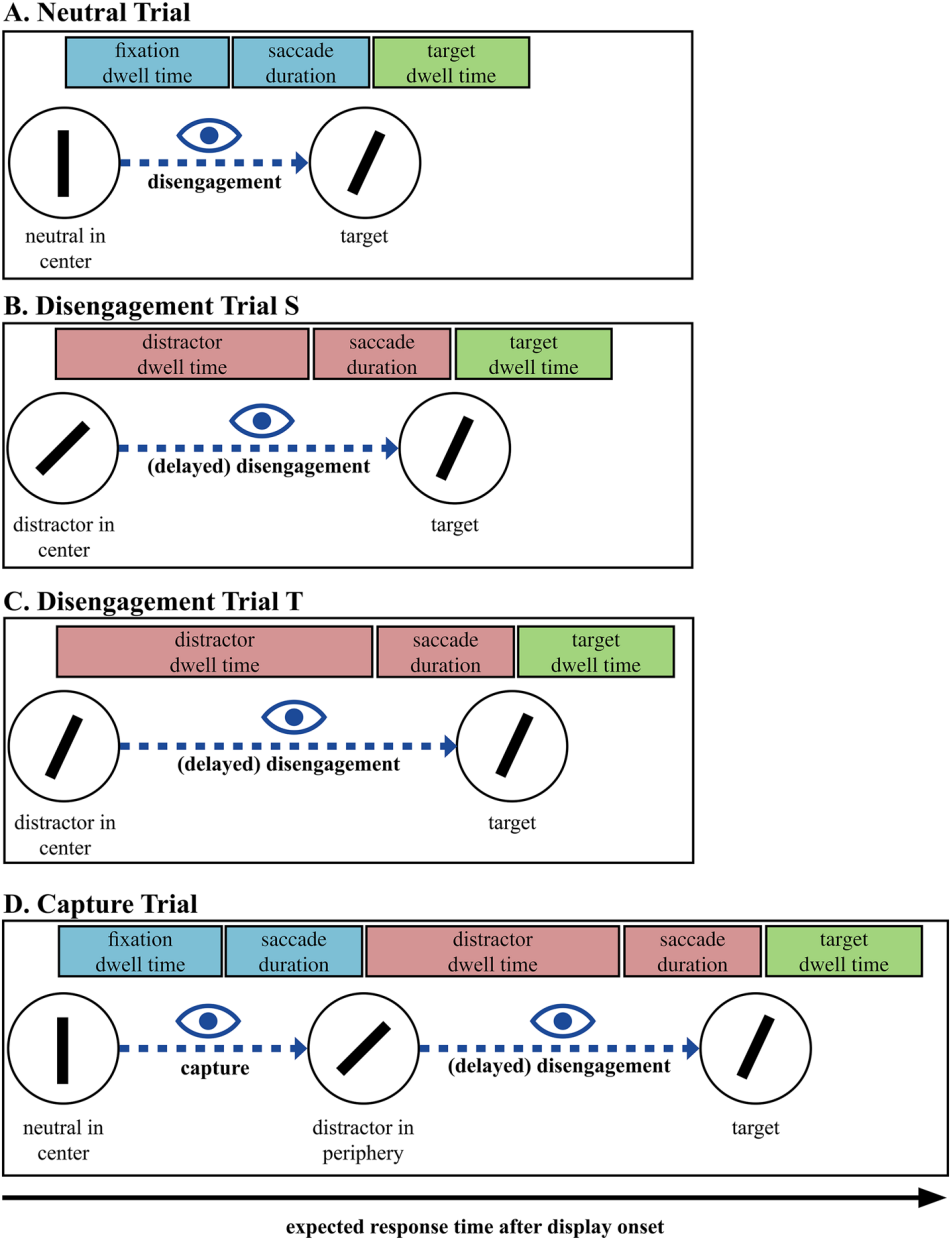
Depicted are the conceptual differences regarding the oculomotor progressions for each of our trial types. The further to the right the target is in the figure, the longer is the expected response time in the respective trials. Please note Fig. 2 to see how the individual visual displays have looked like. We used four different trial types: (**A**) Neutral trials: no distractor was presented and attention can directly be allocated from the fixation item in the center (where participants fixated at the start of each trial) to the target item in the periphery, (**B**) Disengagement trials S: a distractor item that looked like the salient distractor item was presented in the center; (**C**) Disengagement trials T: a distractor item that looked like the target item was presented in the center, also; (**D**) capture trials: the usual capture trials in which attention (often) gets captured by the salient distractor item in the periphery at first and is subsequently allocated to the target item.

**Figure 2:**
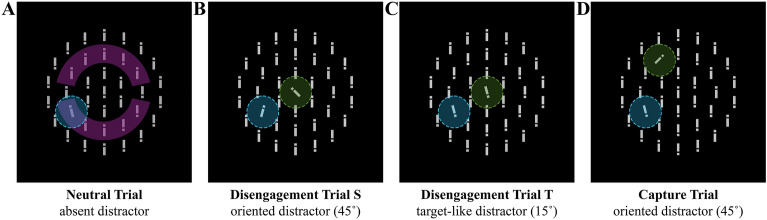
Examples of all possible trials for the neutral trial with no distractor (**A**), the disengagement trial with the distractor oriented in the center (**B**), the disengagement trial with the distractor target-like in the center (**C**), and the capture trial with the distractor oriented in the periphery (**D**). In all panels, the search target was the 15˚-tilted bar at the 8 o'clock position (blue area on the intermediate circle). The distractor was located in the center or at 11 o'clock (green area). The pink area in Panel A indicates the top and bottom semicircles where the target appeared. Blue and green areas also depict the fixation boundary before the search display onset and the saccade landing boundary around the search target. Note that the colored areas are illustrative and were not shown in the experiment, and the distractor and target were randomly tilted left or right.

Therefore, in the present study, we combined their general attentional capture design with a disengagement-only design and similarly employed eye tracking with the aim of dissociating the capture and disengagement mechanisms underlying distractor dwell time; see Fig. 1 for a schematic illustration (also Fig. 2 in the method section for an original search display). In particular, we used four different trial types: (A) Neutral trials: no distractor was presented, and attention could directly be allocated from the fixation item to the target item; (B) Disengagement trials S: a distractor item that looked like the salient distractor item was presented at the center (where participants fixated at the start of the trial); (C) Disengagement trials T: a distractor item that looked like the target item was at the center, also; (D) capture trials: the usual capture trials in which attention (often) gets captured by the salient distractor item in the periphery at first and is subsequently allocated to the target item. Using eye tracking, we can then estimate the time it takes to disengage the eyes from the distractor items, which are either presented classically in the periphery (including oculomotor capture and subsequent oculomotor disengagement—capture trial) or in the center (only oculomotor disengagement under two conditions – disengagement trial S and T). By comparing the time it takes to disengage the eyes in the capture and disengagement trials, we can judge the proportion of capture and disengagement dynamics in attentional capture concerning the oculomotor mechanisms. Building upon the discourse around goal-driven and stimulus-driven factors in attentional capture, this study can provide a deeper insight by analyzing the individual components of distractor dwell time. The differential time scores can support the idea that both top-down and bottom-up processes concurrently contribute to attentional capture, in line with the contingent involuntary orienting hypothesis and stimulus-driven attention models. Our findings emphasize the uniqueness of these processes, suggesting that a simplistic, all-encompassing measure of distractor dwell time could conceal key differences in the underlying mechanisms of attentional capture.

## Methods

### Participants

A total of 42 students (17 females and 25 males, *M* = 22.70, *SD* = 3.64) of the Bundeswehr University Munich participated in this experiment. All participants had normal or corrected-to-normal vision. They provided informed consent and were given course credit for their participation. This study was reviewed and approved by the Ethics Committee at the Bundeswehr University Munich (reference number: EK UniBw M 23–18). Additionally, we declare that our study was conducted in accordance with the ethical standards outlined by the German Association of Psychology and in the 1964 Helsinki Declaration.

### Apparatus

The experiment was programmed in OpenSesame^60^ with the EyeLink Plugin Version 0.6 on a PC running Linux OS with Ubuntu 20.04 LTS. Eye movements were recorded in noise-reduced medium-lit cabins with an EyeLink 1000 Plus (SR Research, Inc) sampling at a rate of 1 kHz. For recruitment, we used the software ORSEE^61^. The stimuli were displayed on an EIZO color monitor with a screen diagonal of 27 inches and a frame rate of 144 Hz at a resolution of 1920 × 1080 pixels. Button presses were recorded with a standard keyboard. Participants responded by pressing the "x" or "m" keys with their left- or right-hand index finger.

### Stimuli

Stimuli were chosen to be similar to prior studies, as they were shown to be reliable in producing strong distractor interference effects^62–66^, with one bar in the center and 36 bars arranged in three imaginary concentric circles with a radius of 3° (degrees of visual angle), 6°, and 9°, comprising of 6, 12, and 18 bars, respectively on a black background. All bars were 0.375° in width and 2.025° in height and had a 0.188° wide gap randomly located 0.375° from the top or the bottom of the bar. Bars were grey (RGB: 127, 127, 127) and were presented in one of three orientations; see also Fig. 2. (1) Non-targets were oriented vertically. (2) Distractors were oriented by being tilted 45˚ clockwise or counterclockwise (orientation randomly selected for each trial), and (3) targets were oriented by tilting 15˚ clockwise or counterclockwise (orientation randomly selected for each trial).

### Design

Participants completed all four types of trials that were intermixed randomly. Relevant stimuli could either appear at the center fixation location or at a position on the middle circle, specifically, in the top half ranging from 10 o'clock to 2 o'clock or the bottom half from 4 o'clock to 8 o'clock position, which is referred to as the top and bottom semicircle (see Fig. 2). In neutral trials (50% of all trials) the target could appear on the middle circle. In disengagement trials S (12,5% of all trials) a 45°-tilted salient distractor appeared in the center, and the target appeared on the middle circle. In disengagement trials T (12,5% of all trials) a 15°-tilted target-like distractor appeared in the center and the target appeared on the middle circle. In capture trials (25% of all trials) both a salient 45°-tiled distractor and the target appeared on the middle circle, but never at the same location. The rest of the locations were occupied by non-targets. The experiment consisted of 672 trials, subdivided into seven blocks of 96 trials each. Within a condition, the probability of the target and, if applicable, distractor appearing at a position was equal.

### Procedure

The participant's task was to detect whether the target bar was interrupted at the top or the bottom (by a gap). If it was interrupted at the bottom, they had to press the "x" key; if it was interrupted at the top, they had to press the "m" key. Participants were informed of four different conditions in which, in addition to the target, there could also be a distractor in the semicircles or in the center, which they were told to ignore because they were irrelevant to their task. Each trial started as soon as the central white fixation cross (radius: 0.2, width: 0.04) was stably fixated within a (virtual) circular area (radius: 2.5). After a randomly selected duration between 700 and 1100 ms, the search display appeared. Participants were instructed to make an eye movement toward the target and to indicate the position of the gap in the target bar by pressing a key. Also, ignore everything in the center as well as possible distractors in the semicircle. They were instructed to do this as quickly and accurately as possible. The search display was presented until a response was made. If the response was incorrect, the word "ERROR" appeared in the center of the screen for 500 ms. The subsequent trial began with the reappearance of the central fixation cross. After each block of trials, participants received feedback on their average RT and accuracy and were allowed to take a short break.

### Eye-data pre-processing

An eye movement was classified as a saccade if its distance exceeded 0.2° and its velocity reached 30°/s. Trials were included in the analyses if the start of the first saccade was at the fixation circle (center) within 2.5 dva from the center (68.36% of all trials) and if at last saccade landed within 2.5 dva from the target (this was not the case in 2.28% of all trials up to the seventh saccade) before a keypress response was given. A saccade was marked as landed on the distractor if its endpoint was within 2.5 dva of the item center. When the target and distractor were adjacent, their ROIs overlapped slightly. We adopted a conservative approach in such cases, classifying fixations in the overlapping region as target-directed. This method ensures that these trials do not affect distractor-related dwell time calculations in the capture condition, thus addressing potential concerns regarding the adjacency of target and distractor items.

### Human rights statements and informed consent

All procedures followed were in accordance with the ethical standards of the responsible committee on human experimentation (institutional and national) and with the Helsinki Declaration of 1964 and its later amendments. Informed consent was obtained from all participants being included in the study.

### Animal rights

This article does not contain any studies with animal subjects performed by any of the authors.

## Results

We removed trials in which observers responded incorrectly (3.22%). Subsequently, we also removed trials in which they did not respond timely within 1.5 times the inter-quartile range for each trial type separately (8.32%).

### Behavioral effects

**Figure 3:**
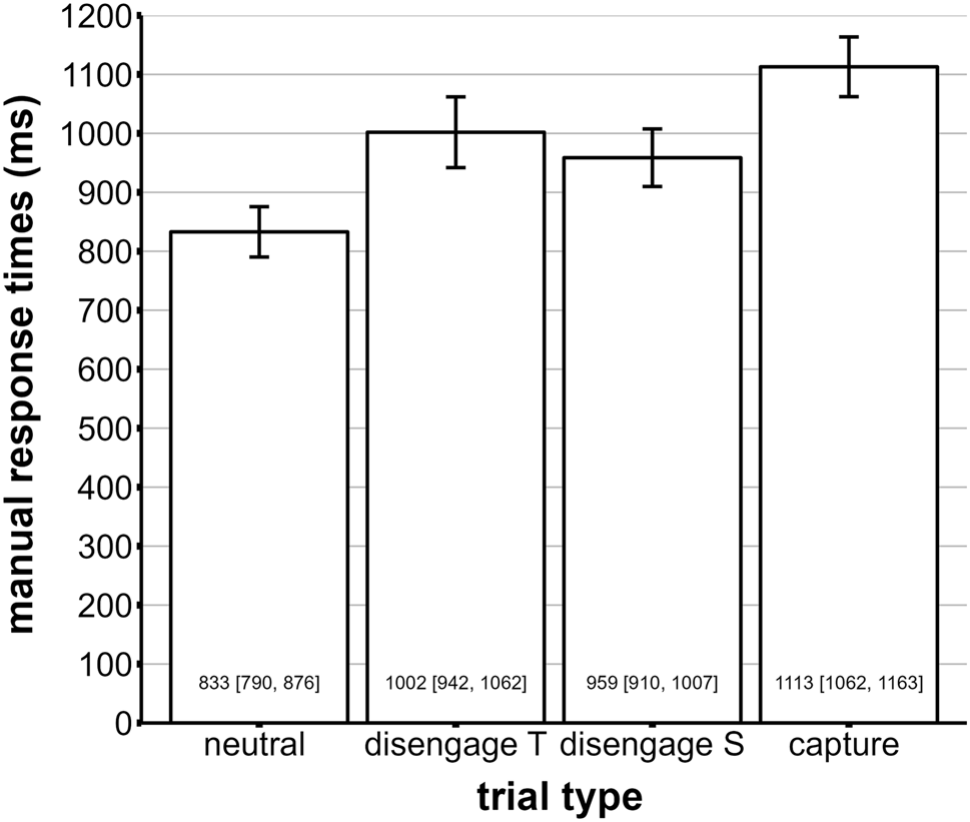
Manual response times depending on the trial type with neutral trials (neutral), disengagement trials with target-like distractors (disengage T), disengagement trials with salient distractors (disengage S), and capture trials (capture). Response times are indicated in ms and the 95% CI.

First, we looked at the manual response times depending on the trial type (neutral trial, capture trial, disengagement trial S and disengagement trial T). A repeated-measures ANOVA revealed a significant main effect of trial type, *F*(2.17, 84.71) = 250.9, *p* < 0.001 (GG-corrected); see also Fig. 3. Paired post-hoc *t*-tests revealed significant differences for all comparisons (all *p*s < 0.001 Holm-corrected). This means that all distractors produced a cost compared to the neutral trial condition.

### Probability of landing on a target

**Figure 4:**
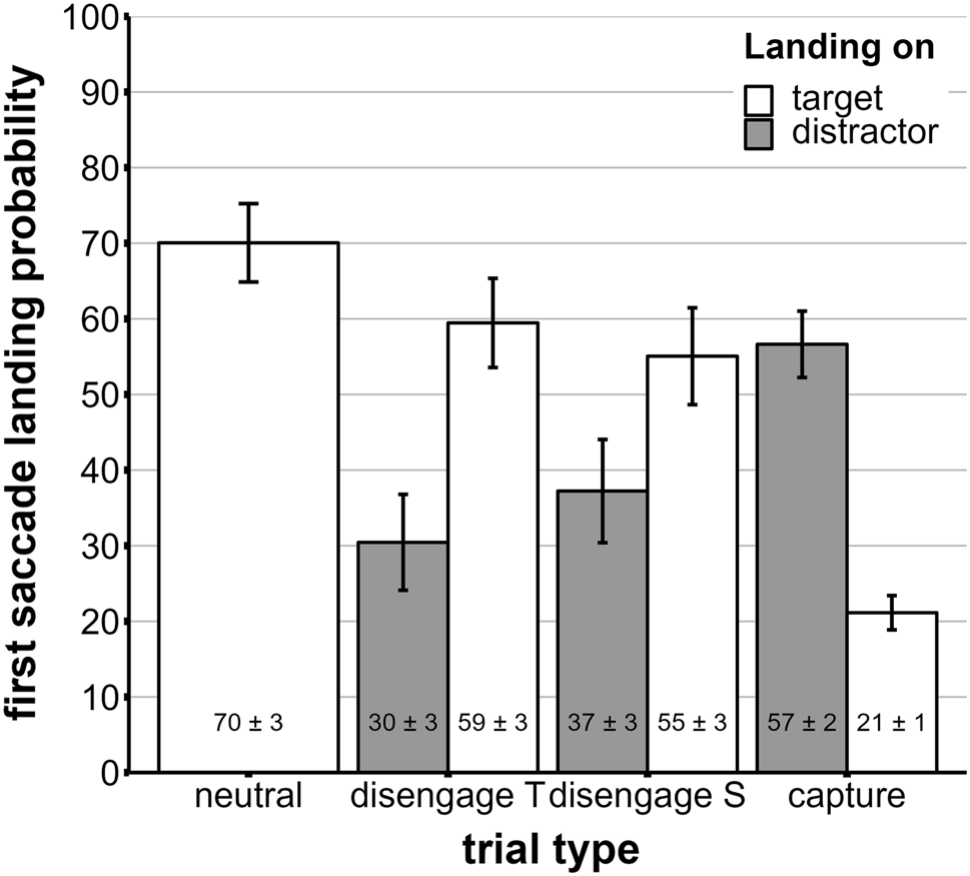
Probability that the first saccade landed on the distractor or target depending on the trial type with neutral trials (neutral), disengagement trials with target-like distractors (disengage T), disengagement trials with salient distractors (disengage S) and capture trials (capture). Probabilities are indicated in % ± SEM.

We then investigated the probability of the first saccade initiated after the display onset landing on the target. A repeated-measures ANOVA with first saccade landing probability depending on trial type (neutral trial, capture trial, disengagement trial S and disengagement trial T) revealed a significant main effect of distractor condition, *F*(2.13, 83.19) = 156.38, *p* < 0.001 (GG-corrected); see Fig. 4. Paired post-hoc *t*-tests revealed significant differences for all comparisons (all *p*s < 0.014). This means the probability that the first saccade landed on the target was reduced in all distractor conditions.

Analogously, we tested the probability that the first saccade landed on the distractor when it was present (as some first saccades landed on other items, this is not a direct reverse analysis of the former). A repeated-measures ANOVA with first saccade landing probability depending on trial type (capture trial, disengagement trial S and disengagement trial T) revealed a significant main effect of trial type, *F*(1.22, 47.5) = 21.46, *p* < 0.001 (GG-corrected) ); see also Fig. 4. Paired post-hoc t-tests revealed significant differences for all comparisons (all *p*s < 0.001). This means the probability that the first saccade landed on the distractor differed between all trial types. Please note that during the disengage and capture trials, the first fixations that did not land on either the target or the distractor could have been directed towards any non-target item in the periphery or may represent refixations on the central neutral item (for capture trials). In the case of central distractors (for disengage S and T trials), first saccades landing on the distractor represent refixations of the central item.

### Dwell times

**Figure 5:**
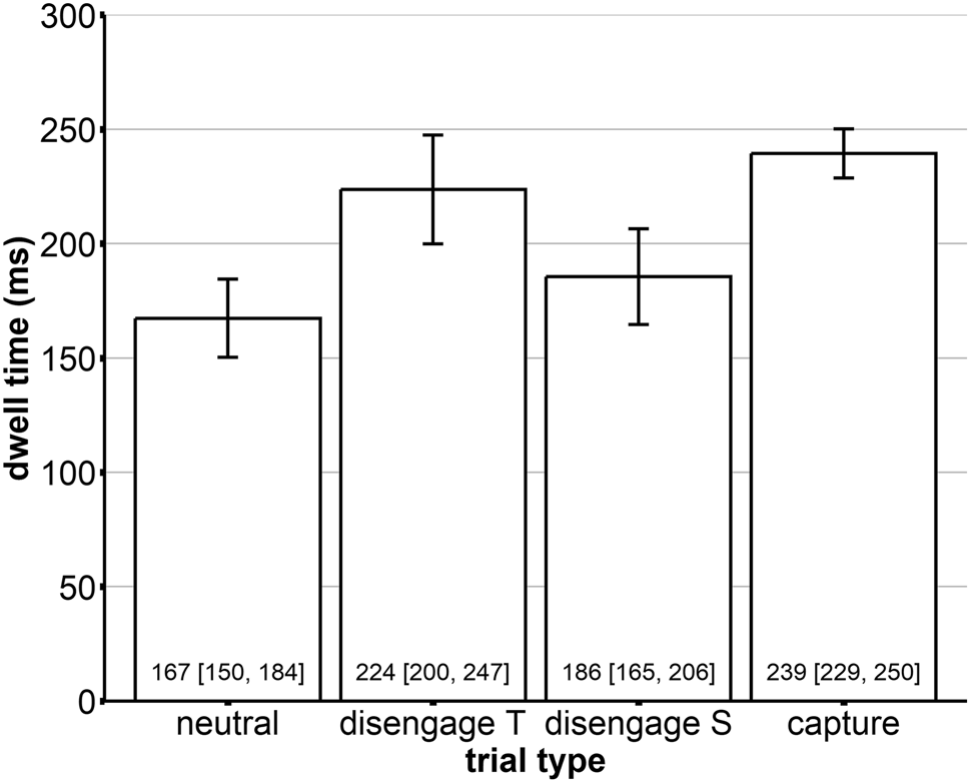
Dwell times (ms) on the distractors in the three distractor conditions, i.e., disengagement trials with target-like distractors (disengage T), disengagement trials with salient distractors (disengage S), and capture trials with salient distractors in the periphery (capture), and dwell times on the central neutral item as a comparison. The error bars indicate the 95% confidence intervals.

**Figure 6:**
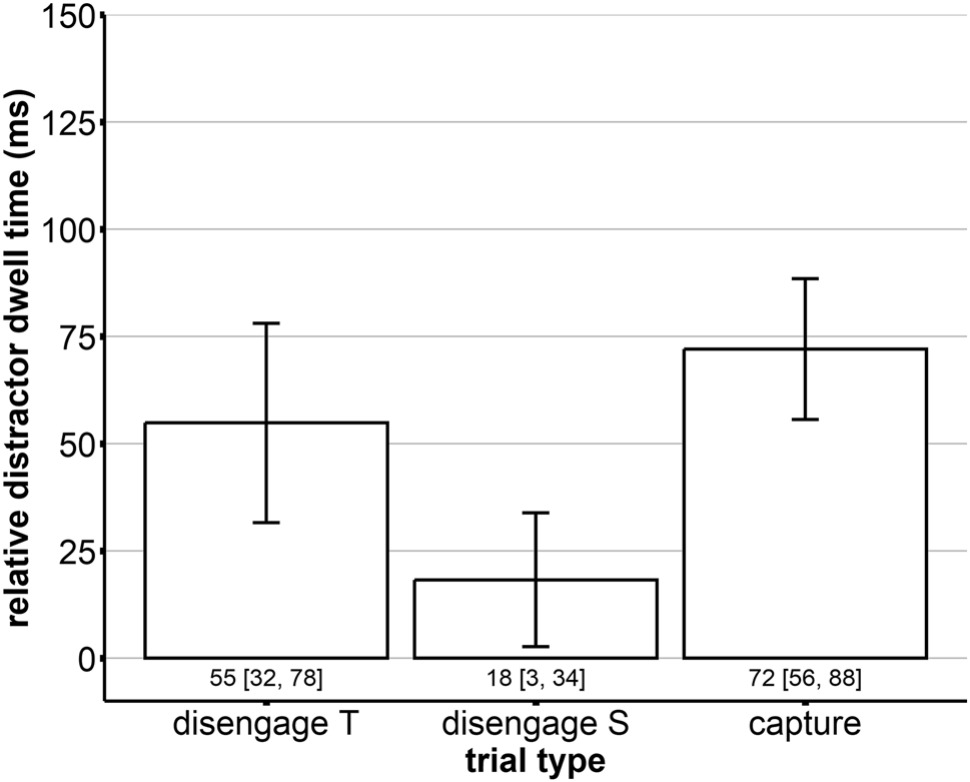
Distractor dwell times (ms) relative to the neutral fixation item (difference) as a function of trial type with disengagement trials with target-like distractors (disengage T), disengagement trials with salient distractors (disengage S), and capture trials with salient distractors in the periphery (capture). The error bars indicated 95% confidence intervals.

To address the quantification of oculomotor capture and disengagement contributions to the distractor dwell time, we investigate the mean dwell times of the distractors. In the first step, we investigate the dwell times ensuing on items after the first saccade, as this has been done in similar studies. A repeated-measures ANOVA with the factor trial type (disengagement trial S, disengagement trial T, capture trial) revealed no significant main effect of trial type, *F*(1.74, 67.94) = 2.29, *p* = 0.116 (GG-corrected). This means we could not observe differential first-saccade dwell times depending on the trial type. However, many observers performed small correctional eye movements that remained on the fixated item. Therefore, in the second step, we computed the total distractor dwell time from the first fixation of a distractor until the start of the first saccade (maximally fifth) that landed on a different item (most often the target) and as a baseline comparison, included dwell times on neutral center items. A repeated-measures ANOVA with the factor trial type (neutral, disengagement trial S, disengagement trial T, capture trial) revealed a significant main effect, *F*(2.53, 96.1) = 19.6, *p* < 0.001, see Fig. 5. In post-hoc t-tests, we found that comparisons with the neutral condition (dwell time: 167 ms) were significant for all three distractor conditions (all *ps* < 0.027), meaning that all distractors produced a delay in disengagement compared to the neutral items, see Fig. 6.

In order to now differentiate between capture and disengagement contributions to attentional capture, we calculated the differences between the mean dwell times on the salient distractor for each observer separately; that is, the *oculomotor disengagement* cost was calculated by subtracting the dwell times in the neutral baseline from the dwell times of the disengagement S condition (resulting in 18 ms), and the *oculomotor capture* cost was calculated by subtracting the dwell times in the disengagement S condition from the dwell times of the capture condition (resulting in 54 ms). We used the "disengagement S" condition when computing the disengagement costs. This condition employs a perceptually identical distractor to the one presented in the "capture" condition, yet it does not share any features with the target. The aim was to isolate and measure the cost associated with disengaging from a non-target distractor without the confounding influence of distractor-target similarity. By contrast, the "disengagement T" condition introduces a distractor resembling the target, which could inflate the dwell time due to additional processing demands needed to differentiate between target-like distractors and actual targets. Hence, using the "disengagement S" condition for our calculations offers a more accurate representation of the pure disengagement costs.

## Discussion

In the present article, we examined how much oculomotor capture and disengagement each contributed to dwell times on distracting items in visual search. In order to do this, we collected manual response times and eye movements in a new combined capture-disengagement paradigm that incorporates elements from delayed disengagement as well as additional-singleton paradigms.

In the present study, we varied the distractor's location to invoke differential capture and disengagement effects. The fastest response times were observed when no distractor was presented (*M* = 833 ms), followed by salient distractors (45°) appearing at the center fixation location (disengagement trials S, *M* = 959 ms), followed by target-like distractors (15°) appearing at the center fixation location (disengagement trials T, *M* = 1002 ms), and salient distractors (45°) appearing at possible target locations in the semicircle (capture trials, *M* = 1113 ms, see Fig. 3). Recent studies suggest that features of the target and distractor, as well as their similarity, differentially affect capture and disengagement^41,45,59^. A salient distractor increases dwell times from 80–90 ms to 120 ms, whereas capture and disengagement could influence this more or less. Born et al.^59^ argued that capture was influenced by bottom-up and disengagement by top-down processes. They argue that once attention was captured, the signal was sustained and thus could be modulated from top to bottom by higher-level cognitive areas. Results from Stefani and colleagues^58^ support the hypothesis of top-down influences from distractors. In various search displays, they demonstrated that an irrelevant stimulus similar to the search target significantly increased disengagement (~ 20 – 35 ms). However, the extent to which capture and disengagement influence dwell time is still unclear.

In our experiment, we considered dwell times on the distractor. We could show that capture trials led to the highest distractor dwell times (*M* = 72 ms), followed by target-like distractors appearing at the center fixation location (*M* = 55 ms) and salient distractors appearing at the center fixation location (*M* = 18 ms; see Fig. 6). Thus, the capture effect aligns with earlier studies showing that distractors appearing at irrelevant locations increase dwell times^59^. Also, for the irrelevant center location, it is plausible that distractors sharing features with the target (here: all features except for their location) delay the oculomotor disengagement from them, thereby leading to higher interference^56–59^. Notably, while salient distractors appearing at the center fixation location produced less interference than target-like distractors appearing at the center fixation location, they still produced a sizable interference effect. So, overall, the present paradigm is suitable for inducing distractor interference effects in all critical locations. In other words, capture trials concerning capture and disengagement were slower than pure disengagement trials (for both target-like and salient distractors). Thus, our results are in line with the previously established oculomotor capture^45,59^ and delayed oculomotor disengagement effects^58,67^. An important aspect to keep in mind while interpreting our 'oculomotor capture cost' results is the intricate dynamics of saccadic programming. Our calculation represents a scenario with concurrent saccades directed toward both the distractor and the target, creating a form of competition on a priority map. Despite the distractor often 'winning' this competition for the first saccade, the broader implications of this competition and its potential modulation by various factors are nuanced. The 'oculomotor capture cost' can also be affected by these variables. We delve deeper into these complex interactions in the sections that follow.

The investigation's central aim was to quantify the relative contributions of capture and disengagement to the attentional capture effect. In the present paradigm, we found that oculomotor disengagement costs can be estimated at around 18 ms, while oculomotor capture costs can be estimated at around 54 ms. Thus, disengagement from the distractor is comparable to Stefani and colleagues^58^, which argued for top-down processes on the distractor and reinforced Born et al.'s^59^ conjecture. The similarities in findings underpin the notion that top-down processes significantly influence disengagement.

Regarding the dwell time in capture trials, we found that while oculomotor capture significantly contributes to the dwell time on distractors, it does not account for the entirety of the observed dwell time. This suggests that stimulus properties do not solely drive the dwell time in capture trials. Additionally, the perfect predictability of distractor location in the disengagement trials (always appearing at the center) might have reduced disengagement time, potentially causing an underestimation of the 'true' oculomotor disengagement time that would be relevant for peripheral distractors. Thus, the oculomotor disengagement costs are potentially even higher. Nevertheless, we acknowledge that our understanding of these processes and their contribution to dwell time remains incomplete. Additional research, including studies exploring how various types of distractors and different visual search conditions affect these processes, will help further refine our understanding of the dynamics of oculomotor capture and disengagement.

## Conclusion

In visual search, items that show similarities to the search target capture attention and cause response times to increase. So far, the features of a distracting item that affects search have been investigated, but not the relative contributions of the oculomotor processes underlying the distractor-related dwell time. We used a combined experimental design invoking oculomotor capture and disengagement separately. We could show that only two-thirds of distractor-related dwell time can be explained by oculomotor capture of attention. One-third is explained by the (inability) of oculomotor disengagement, which has been neglected or underestimated in previous studies. Therefore, both stimuli-driven (oculomotor capture) and goal-directed (oculomotor disengagement) processes play a significant role in the time we spend scrutinizing distracting items in visual search.

## Data Availability

The datasets generated during and/or analysed during the current study are available in the OSF repository, https://osf.io/c8fxj/.

## Code availability

The code is available in the OSF repository, https://osf.io/c8fxj/.
